# Pharmacokinetics, Safety, and Tolerability of Intravenous Durlobactam and Sulbactam in Subjects with Renal Impairment and Healthy Matched Control Subjects

**DOI:** 10.1128/AAC.00794-19

**Published:** 2019-08-23

**Authors:** John O’Donnell, Richard A. Preston, Grigor Mamikonyan, Emily Stone, Robin Isaacs

**Affiliations:** a Entasis Therapeutics, Waltham, Massachusetts, USA; b Division of Clinical Pharmacology Phase I Unit, Miller School of Medicine University of Miami, Miami, Florida, USA; c Clinartis LLC, Hollywood, Florida, USA; d Spero Therapeutics, Cambridge, Massachusetts, USA

**Keywords:** ETX2514, pharmacokinetics, renal impairment, safety, sulbactam

## Abstract

Sulbactam-durlobactam is being developed for the treatment of infections caused by Acinetobacter baumannii, including those caused by multidrug- and carbapenem-resistant isolates. This was a phase 1 study to evaluate the effects of various degrees of renal impairment, including subjects with end-stage renal disease (ESRD) on hemodialysis (HD), on the pharmacokinetics and safety profile of durlobactam (also known as ETX2514) and sulbactam after single intravenous (i.v.) dose administration. For healthy subjects and those with mild or moderate renal impairment (RI), single 1,000-mg doses each of durlobactam and sulbactam via a 3-h i.v. infusion were administered, and for severe renal impairment, 500-mg doses were administered. For subjects with ESRD and HD, 500-mg i.v. doses each of durlobactam and sulbactam were administered post-HD and pre-HD, with a 1-week washout between doses. Among 34 subjects, decreasing renal function increased systemic exposure (peak plasma concentration [*C*_max_] and area under the concentration-time curve [AUC]) to durlobactam and sulbactam in a generally linear manner. In healthy subjects and in those with mild or moderate renal impairment, the majority of durlobactam and sulbactam was excreted in the urine, while approximately 40% or less was excreted in urine in subjects with severe renal impairment or ESRD. In subjects with ESRD, hemodialysis was effective at removing both durlobactam and sulbactam from plasma. Renal impairment had no effect of the safety/tolerability profile of durlobactam and sulbactam. In summary, RI and ESRD had a predictable effect on the pharmacokinetic (PK) profile of durlobactam and sulbactam with no adverse effects on the safety/tolerability profile. Durlobactam and sulbactam are cleared to a similar extent by renal elimination and are impacted similarly by renal impairment. The results from this study have been used with population PK modeling and nonclinically derived PK/PD (pharmacodynamic) exposure targets to establish dosage recommendations for durlobactam and sulbactam in patients with various degrees of RI. The dosing regimen of durlobactam-sulbactam will require adjustment in patients with severe renal insufficiency and in those with ESRD.

## Abstract (precis)

Sulbactam-durlobactam is being developed for the treatment of infections caused by Acinetobacter baumannii, including those caused by multidrug- and carbapenem-resistant isolates. This was a phase 1 study to evaluate the effects of various degrees of renal impairment, including subjects with end-stage renal disease (ESRD) on hemodialysis (HD), on the pharmacokinetics and safety profile of durlobactam (also known as ETX2514) and sulbactam after single intravenous (i.v.) dose administration.

## INTRODUCTION

The incidence of serious, multidrug resistant (MDR) infections is on the rise globally; thus, a need exists for new, effective antimicrobials to treat serious infections (1, 2). Acinetobacter baumannii is one of the pathogens identified by the Centers for Disease Control and World Health Organization as a critical priority in need of new treatment options (1, 2). Multidrug resistant A. baumannii is the cause of serious infections that are associated with high rates of morbidity and mortality (3–9).

Durlobactam (also known as ETX2514) is a novel, diazabicyclooctenone β-lactamase inhibitor (BLI) that potently inhibits class A, C, and D β-lactamases (10). Durlobactam exhibits *in vitro* activity against some Enterobacteriaceae but has no significant activity against A. baumannii. Sulbactam is a β-lactam inhibitor with demonstrated activity against A. baumannii; however, increasing resistance has limited its use as monotherapy in recent years (11). Sulbactam-durlobactam is a combination of durlobactam and sulbactam that is being developed for the treatment of infections caused by A. baumannii, including those caused by MDR and carbapenem-resistant isolates. Preclinical studies show potent *in vitro* and *in vivo* activity with sulbactam-durlobactam against A. baumannii (12–14). Phase 1 clinical studies in healthy subjects have characterized the pharmacokinetic (PK) profile of durlobactam after single and multiple ascending doses and in combination with sulbactam and determined plasma and intrapulmonary concentrations of both components (15, 16).

Renal elimination is the major route of elimination for both durlobactam and sulbactam. An important part of the clinical development is to investigate effect of various degrees of renal impairment (RI) on the safety and PK profile of durlobactam and sulbactam to determine the need for dosage adjustment. This phase 1 study investigated the effects of various degrees of RI, including subjects with end-stage renal disease (ESRD) on hemodialysis (HD), on the PK and safety profile of sulbactam-durlobactam after single intravenous (i.v.) dose administration.

## RESULTS

A total of 34 subjects were enrolled, and all completed the study. One subject (ESRD on HD cohort) was excluded from the PK analysis due to anomalously high plasma concentrations; although the explanation for plasma concentrations in this subject is unknown, the concentrations of durlobactam and sulbactam were 20- to 100-fold higher in samples collected during the infusion compared to those from other subjects in the same cohort. The healthy cohort was comparable to other cohorts with RI for baseline characteristics except for estimated glomerular filtration rate (eGFR) and creatinine clearance (CL_CR_) (Table 1).

**TABLE 1:** Baseline patient characteristics

Patient characteristic^*b*^	Normal renal function (*n* = 8)	RI			ESRD (*n* = 6)
		Mild (*n* = 6)	Moderate (*n* = 6)	Severe (*n* = 8)	
Age (yrs)^*a*^	59.6 ± 7.6	64.5 ± 5.8	62.3 ± 8.5	59.9 ± 11.0	51.2 ± 9.7
Male (*n* [%])	5 (62.5)	3 (50.0)	2 (33.3)	5 (62.5)	3 (50.0)
Race (*n* [%])					
White	7 (87.5)	3 (50.0)	5 (83.3)	6 (75.0)	2 (33.3)
Black or African American	1 (12.5)	3 (50.0)	1 (16.7)	2 (25.0)	4 (66.7)
Hispanic or Latino (*n* [%])	4 (50.0)	0	0	4 (50.0)	1 (16.7)
Wt (kg)^*a*^	83.6 ± 9.7	83.2 ± 15.3	93.6 ± 18.7	83.9 ± 8.4	88.5 ± 19.5
BMI (kg/m^2^)^*a*^	28.6 ± 2.8	30.7 ± 5.6	31.9 ± 5.6	30.4 ± 4.4	30.0 ± 5.7
eGFR (ml/min/1.73 m^2^)^*a*^	92.0 ± 11.0	71.8 ± 15.5	42.0 ± 9.0	14.9 ± 6.5	7.7 ± 3.0
CL_CR_ (ml/min)^*a*^	111.2 ± 19.7	79.3 ± 13.8	61.6 ± 12.8	22.4 ± 9.9	13.3 ± 4.9

a Mean ± standard deviation.

b BMI, body mass index; CL_CR_, creatinine clearance; eGFR, estimated glomerular filtration rate.

### Pharmacokinetics.

**FIG 1:**
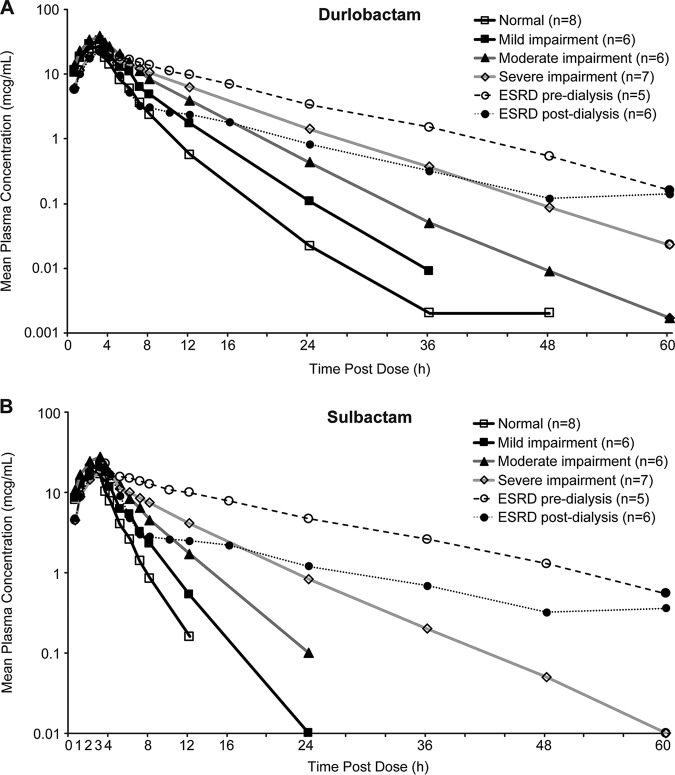
Mean (± standard deviation [SD]) plasma durlobactam (A) and sulbactam (B) concentration versus time profiles by cohort (semi-log).

**(i) Durlobactam.** Mean durlobactam concentration increased with decreasing renal function in healthy subjects, those with RI, and subjects with ESRD (Fig. 1). The median *T*_max_ was approximately 3 h for all treatment groups (Table 2). Half-life ranged from 2.3 to 7.1 h and increased with increasing renal impairment. Geometric mean peak plasma concentration (*C*_max_) ranged from 21.9 to 38.7 μg/ml (Table 2), with dose-normalized values that increased from 0.027 μg/ml/mg in healthy subjects to 0.0572 μg/ml/mg in the first period of the ESRD group. Dose-normalized *C*_max_ for period 2 in ESRD subjects was 0.0439 μg/ml/mg, which was between those of the moderate and severe RI groups. *C*_max_ was consistently lower in period 2 than in period 1 for ESRD subjects, which was likely due to greater fluid volume allowing for greater dilution of drug in ESRD subjects receiving durlobactam and sulbactam infusions prior to their regularly scheduled dialysis session. Geometric mean area under the concentration-time curve from time 0 to infinity (AUC_0–inf_) values ranged from 110 to 280 h · μg/ml (Table 2). Dose-normalized AUC_0–inf_ followed similar trends as those described for dose-normalized *C*_max_. Estimates of clearance (CL) and terminal phase volume of distribution (*V*_z_) varied by treatment group and tended to decrease as renal impairment increased (Table 2).

**TABLE 2:** Geometric mean (% coefficient of variation) PK parameters for durlobactam

PK parameter^*d*^	Normal renal function (*n* = 8)	RI			ESRD period	
		Mild (*n* = 6)	Moderate (*n* = 6)	Severe (*n* = 7)	1 (*n* = 5)	2 (*n* = 6)
Dose (mg)	1,000	1,000	1,000	500	500	500
*C*_max_ (μg/ml)	27.0 (19.3)	33.3 (18.9)	38.7 (20.9)	25.5 (21.4)	28.6 (27.5)	21.9 (22.7)
*T*_max_ (h)^*a*^	3.0 (2.0–3.1)	3.0 (2.0–3.0)	3.0 (2.0–3.2)	3.0 (3.0–4.0)	3.0 (2.0–3.5)	3.0 (3.0–4.0)
Half-life (h)	2.3 (4.3)^*b*^	3.0 (14.8)	3.8 (21.9)	5.4 (20.5)	7.1 (36.6)	7.1 (32.7)^*c*^
AUC_0–last_ (h · μg/ml)	109 (16.7)	151 (19.1)	209 (33.2)	202 (24.0)	278 (45.2)	123 (28.5)
AUC_0–inf_ (h · μg/ml)	110 (17.6)^*b*^	151 (19.1)	209 (33.2)	202 (24.0)	280 (45.6)	123 (32.1)^*c*^
CL (ml/h)	9,090 (17.6)^*b*^	6,640 (19.1)	4,790 (33.2)	2,470 (24.2)	1,780 (45.6)	4,070 (32.1)^*c*^
*V*_z_ (ml)	30,600 (15.3)^*b*^	28,500 (20.1)	26,000 (12.6)	19,200 (12.3)	18,300 (13.0)	41,900 (21.9)^*c*^
CL_R_ or CL_D_ (ml/h)	6,080 (22.0)	5,040 (20.8)	3,420 (35.6)	947 (53.8)		7,600 (24.2)
Fe_u_/Fe_HD_	0.66 (18.3)	0.78 (7.1)	0.72 (14.8)	0.38 (29.1)		0.33 (35.6)

a Median (range).

b *n* = 7.

c *n* = 5.

d AUC_0–last_ or AUC_0–inf_, area under the concentration-time curve, time 0 to last or time 0 to infinity; CL, clearance; CL_D_, estimated hemodialysis recovery clearance; CL_R_, renal clearance; *C*_max_, peak plasma concentration; Fe_HD_, fraction of dose removed by hemodialysis; Fe_u_, fraction of the dose renally eliminated; *T*_max_, time to peak plasma concentration; *V*_z_, terminal volume of distribution.

Renal clearance (CL_R_) decreased as renal impairment increased, with geometric mean values that ranged from 6,080 ml/h in healthy subjects to 947 ml/h in severe RI subjects. For healthy subjects and those with mild or moderate RI, renal clearance values represented 66% to 76% of total clearance. Approximately 70% of the durlobactam dose was eliminated in urine for healthy subjects and those with mild or moderate RI, with most excretion occurring within 24 h of dosing. For severe RI, renal clearance represented 38% of plasma clearance. For ESRD subjects on HD, approximately 33% of the 500-mg durlobactam dose was excreted in dialysate, which was similar to the amount excreted in urine for severe RI subjects. This corresponded to a geometric mean estimated hemodialysis recovery clearance (CL_D_-recovery) of 7,600 ml/h, which exceeded the reported total plasma clearance (CL). CL_D_-recovery exceeded the calculated renal clearance for healthy subjects, suggesting that dialysis effectively removed durlobactam from plasma. The geometric mean for dialysis-mediated clearance calculated from the difference between arterial and venous plasma concentrations during dialysis (CL_HD_) was 12,400 ml/h.

**(ii) Sulbactam.** Similarly to durlobactam, mean sulbactam concentration increased with decreasing renal function in healthy subjects, those with RI, and subjects with ESRD (Fig. 1). The median sulbactam *T*_max_ was 3 h for all treatment groups, and geometric mean *C*_max_ values ranged from 17.0 to 23.7 μg/ml (Table 3). Dose-normalized *C*_max_ values increased from 0.0170 μg/ml/mg in healthy subjects to 0.0473 μg/ml/mg in ESRD subjects dosed after dialysis. Geometric mean AUC_0–inf_ values ranged from 63.0 to 288 h · μg/ml across all treatment groups (Table 3), with similar trends as *C*_max_ for dose-normalized AUC_0–inf_. CL decreased as renal impairment increased, ranging from 1,740 ml/h in ESRD subjects to 15,900 ml/h in healthy subjects, and half-life ranged from 1.8 to 10.0 h with increasing renal impairment. Geometric mean values for renal clearance tended to decrease as renal impairment increased, ranging from 10,300 ml/h in healthy subjects to 1,550 ml/h in those with severe RI. Approximately 65% to 93% of the sulbactam dose was eliminated in urine for healthy subjects and those with mild or moderate RI, with most excretion occurring within 24 h of dosing. For subjects with severe RI, approximately 42% of the dose was excreted in urine. For subjects with ESRD, approximately 41% of the 500-mg dose was excreted in dialysate during a dialysis period of approximately 4 h, which corresponded to a geometric mean CL_D_-recovery of 10,500 ml/h, similar to the renal clearance for healthy and mild RI subjects. The geometric mean for dialysis-mediated clearance was 12,900 ml/h.

**TABLE 3:** Geometric mean (% coefficient of variation) PK parameters for sulbactam

PK parameter^*c*^	Normal renal function (*n* = 8)	RI			ESRD period	
		Mild (*n* = 6)	Moderate (*n* = 6)	Severe (*n* = 7)	1 (*n* = 5)	2 (*n* = 6)
Dose (mg)	1,000	1,000	1,000	500	500	500
*C*_max_ (μg/ml)	17.0 (17.1)	22.1 (25.3)	27.1 (28.9)	20.2 (26.0)	23.7 (32.8)	20.1 (23.2)
*T*_max_ (h)^*a*^	3.0 (2.0–3.0)	3.0 (2.0–3.0)	3.0 (2.0–3.2)	3.0 (3.0–4.0)	3.0 (2.0–3.5)	3.0 (3.0–4.0)
Half-life (h)	1.8 (37.9)	2.0 (15.1)	3.0 (43.6)	4.6 (34.0)	9.2 (52.8)	10.0 (51.6)
AUC_0–last_ (h · μg/ml)	62.8 (14.7)	85.3 (28.7)	126 (42.3)	136 (40.2)	280 (71.5)	127 (37.1)
AUC_0–inf_ (h · μg/ml)	63.0 (14.7)	85.8 (28.1)	126 (42.0)	136 (40.2)	288 (73.9)	131 (43.1)^*b*^
CL (ml/h)	15,900 (14.7)	11,700 (28.1)	7,930 (42.0)	3,660 (40.4)	1,740 (73.9)	3,820 (43.1)^*b*^
*V*_z_ (ml)	40,100 (38.3)	32,800 (20.4)	33,700 (18.3)	25,000 (15.8)	23,100 (19.6)	55,000 (20.0)^*b*^
CL_R_ or CL_D_ (ml/h)	10,300 (22.6)	10,900 (26.6)	6,950 (43.6)	1,550 (69.6)		10,500 (24.7)
Fe_u_/Fe_HD_	0.65 (19.3)	0.93 (3.8)	0.88 (16.8)	0.42 (26.3)		0.41 (40.5)

a Median (range).

b *n* = 5.

c AUC_0–last_ or AUC_0–inf_, area under the concentration-time curve, time 0 to last or time 0 to infinity; CL, clearance; CL_D_, estimated hemodialysis recovery clearance; CL_R_, renal clearance; *C*_max_, peak plasma concentration; Fe_HD_, fraction of dose removed by hemodialysis; Fe_u_, fraction of the dose renally eliminated; *T*_max_, time to peak plasma concentration; *V*_z_, terminal volume of distribution.

For durlobactam, geometric coefficient of variation (CV%) values were generally under 40% for PK parameters. Intersubject variability was generally higher for sulbactam than for durlobactam; geometric CV% values were under 50% for PK parameters. Variability tended to increase by cohort as renal function decreased for both durlobactam and sulbactam.

### Statistical comparisons of pharmacokinetics.

The analysis of the effect of RI on durlobactam and sulbactam plasma PK parameters by analysis of variance (ANOVA) is summarized for durlobactam and sulbactam (Table S1). All cohorts with RI exhibited higher exposure to durlobactam and sulbactam than did healthy subjects (matched to cohort 4) compared using ANOVA of ln-transformed dose-normalized *C*_max_, AUC from time 0 to last (AUC_0–last_), and AUC_0–inf_. For ESRD subjects, durlobactam and sulbactam exposure (as assessed by ANOVA of ln-transformed *C*_max_/dose, AUC_0–last_/dose, and AUC_0–inf_/dose) was lower when administration was completed approximately 1 h prior to dialysis compared to that when durlobactam and sulbactam were administered after the end of a dialysis session (Table S2).

**FIG 2:**
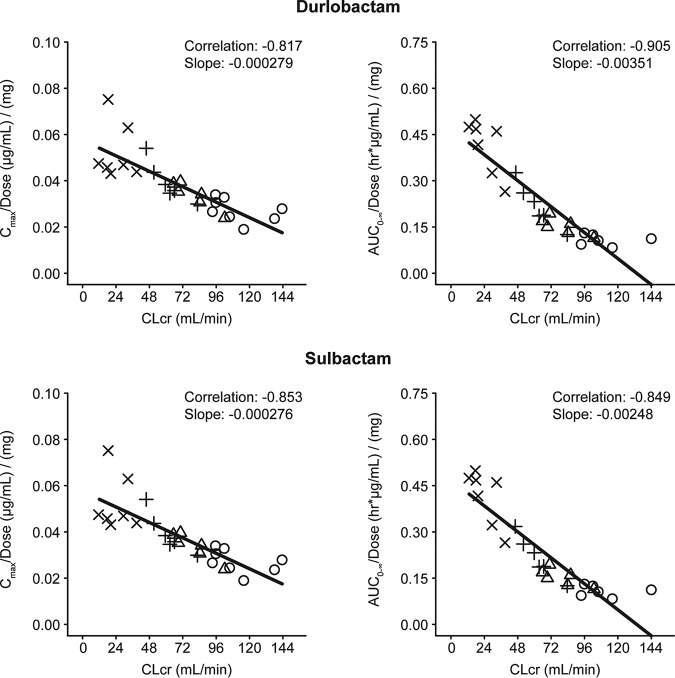
Linear regression analysis for dose-normalized durlobactam and sulbactam exposure (*C*_max_/dose and AUC_0–inf_/dose) versus CL_CR_. Open circle, normal renal function; open triangle, mild RI; +, moderate RI; X, severe RI.

Regression analyses of durlobactam and sulbactam exposure (*C*_max_/dose, AUC_0–inf_/dose) versus CL_CR_ in cohorts 1 through 4 demonstrated strong correlations with negative slopes (Fig. 2). Results for AUC_0–last_ were consistent with those for AUC_0–inf_. Results for eGFR were similar to those for CL_CR_ for exposure with both durlobactam and sulbactam (data not shown). Correlations of exposure with eGFR also suggested that dose-normalized exposure may be predictable based on subject eGFR values across a given linear dose range.

### Safety/tolerability.

Six (17.6%) subjects experienced 9 adverse events (AEs), namely, 3 (50.0%) subjects with moderate RI, 1 (12.5%) with severe RI, and 2 (33.3%) with ESRD on HD (Table 4). No healthy subjects or subjects with mild RI experienced an AE. Six of 9 AEs were mild in severity, and 3 AEs were moderate in severity. One (16.7%) subject on ESRD on HD reported an AE of nausea that was considered to be related to study treatment and resolved the same day without any intervention. No clinically relevant changes in clinical laboratory, electrocardiogram (ECG), vital signs, or physical examination were observed. In general, the safety profile observed in this study was consistent with that seen in the development program thus far.

**TABLE 4:** Incidence of adverse events with durlobactam and sulbactam

Adverse event	No. (%) of subjects/number of events				
	Normal (*n* = 8)	RI			ESRD (*n* = 6)
		Mild (*n* = 6)	Moderate (*n* = 6)	Severe (*n* = 8)	
Any AE^*a*^	0	0	3 (50.0)/5	1 (12.5)/2	2 (33.3)/2
Dizziness	0	0	1 (16.7)	0	0
Epistaxis	0	0	0	1 (12.5)	0
Fall	0	0	1 (16.7)	0	0
Foot fracture	0	0	1 (16.7)	0	0
Infusion site extravasation	0	0	1 (16.7)	0	0
Mucosal dryness	0	0	0	1 (12.5)	0
Nausea	0	0	1 (16.7)	0	1 (16.7)
Viral upper respiratory infection	0	0	0	0	1 (16.7)

a AE, adverse event.

## DISCUSSION

The results from this study showed that with decreasing renal function, systemic exposure (*C*_max_ and AUC) to durlobactam and sulbactam increased in a generally linear manner, which is consistent with renal elimination as the primary clearance mechanism for both compounds. In healthy subjects and those with mild or moderate RI, the majority of durlobactam and sulbactam was excreted in the urine, while approximately 40% or less was excreted in urine in subjects with severe RI or ESRD. Importantly, in subjects with ESRD, hemodialysis was effective at removing both durlobactam and sulbactam from plasma. As a result, dosage adjustment is needed in patients with severe RI (eGFR of <30 ml/min/1.73 m^2^) or those with ESRD on HD. Based on these results, CL_CR_ and eGFR can be used to predict dose-normalized exposure in subjects with RI and provide guidance for dosage adjustment in those with RI. Importantly, RI had no effect of the safety/tolerability profile of durlobactam or sulbactam.

The PK parameters observed in healthy subjects after a single dose of durlobactam (1 g) in this study were comparable to results previously reported with durlobactam (1 g) in other studies of healthy subjects with normal renal function (15, 16). Furthermore, PK parameters after a single dose of sulbactam (1 g) in healthy subjects in this study were generally similar to those in other studies of healthy subjects with normal renal function (16–18).

The observation of increasing RI resulting in increased systemic exposure to durlobactam and sulbactam has also been observed with other BLIs (19–21), as well as with sulbactam (17, 18), and is likely associated with significant renal elimination of these compounds. In addition, the effect of RI on PK parameter for sulbactam among subjects in this study was similar to results from previous studies of sulbactam (17, 18).

In summary, RI and ESRD had a predictable effect on the PK profile of durlobactam and sulbactam, with no adverse effects on the safety/tolerability profile. Both durlobactam and sulbactam are cleared to a similar extent by renal elimination and are impacted similarly by renal impairment. As such, the ratio of durlobactam and sulbactam in the combination product sulbactam-durlobactam can be maintained regardless of the degree of RI and supports the development of a fixed dose combination product. These results have been used with population PK modeling and nonclinically derived PK/PD exposure targets to establish dosage recommendations for durlobactam and sulbactam in patients with various degrees of RI (22). The dosing regimen of sulbactam-durlobactam will require adjustment in patients with severe renal insufficiency and those with ESRD, as well as in patients with augmented renal clearance.

Multidrug-resistant infections due to Acinetobacter are often treated with polymyxins like colistin, which carry a substantial risk of nephrotoxicity. Given the severity of sepsis in such patients, compromised renal function is often present at baseline, and developing an effective therapy that is less toxic, such as sulbactam-durlobactam, could be critical to improving outcomes in this difficult-to-treat population. A phase 3 study is currently ongoing to evaluate efficacy and safety of i.v. durlobactam and sulbactam (1 g of each drug infused concurrently over 3 h every 6 h), along with imipenem-cilastatin, in patients with serious infections due to Acinetobacter spp., including those with hospital-acquired or ventilator-associated bacterial pneumonia and bloodstream infections. The RI-based dosing recommendations for durlobactam and sulbactam will be utilized in this study.

## MATERIALS AND METHODS

The study enrolled subjects at three clinical sites in the United States between September 2017 and May 2018. The study was conducted in accordance with the Declaration of Helsinki and Good Clinical Practices. The study protocol and amendments were approved by an Institutional Review Board for each clinical site, and all subjects provided written informed consent prior to any study procedure.

### Study design.

This was a phase 1, open-label, nonrandomized study to evaluate the PK, safety, and tolerability of a single concurrent i.v. infusion of durlobactam (500 or 1,000 mg) and sulbactam (500 or 1,000 mg) in healthy subjects with normal renal function (CL_CR_, ≥90 ml/min), subjects with mild, moderate, or severe RI (eGFR, ≥60 to <90, ≥30 to <60, or <30 ml/min/1.73 m^2^, respectively), and subjects with ESRD on HD. The Cockcroft-Gault equation was used to calculate creatinine clearance (23), and eGFR was calculated using the modification of diet in renal disease (MDRD) equation (24).

### Subject selection.

Male or female subjects ages 18 to 75 years with a body mass index (BMI) of ≥18 to ≤40 kg/m^2^ were eligible. Females were not pregnant or lactating and were either postmenopausal, surgically sterile, or agreed to use an acceptable form of contraception throughout the study. Male subjects agreed to use acceptable contraception. Subjects were considered healthy based on medical and surgical history, physical examination, 12-lead ECG, and clinical laboratory testing with an estimated CL_CR_ of ≥90 ml/min. Healthy subjects were matched to subjects with severe RI for gender, body mass index (BMI ± 20%), and age (±10 years). Healthy subjects were nonsmokers for at least 30 days before screening and were not taking any prescription or over-the-counter medications. Subjects with RI and ESRD were considered clinically stable based on medical and surgical history, physical examination, ECG, and clinical laboratory testing, with aspartate aminotransferase (AST), alanine aminotransferase (ALT), and total bilirubin levels within 1.5× the upper limit of normal (ULN) at screening. Subjects with ESRD were on a stable HD regimen, defined as time for dialysis clearance of urea/volume of distribution for urea (*k*_t_/*V*) of ≥1.2 within 3 months prior to screening with at least 3 HD treatments per week. Subjects with RI and ESRD were allowed to take chronic medications and were permitted to smoke ≤10 cigarettes/day.

Subjects were excluded for any hypersensitivity to β-lactams; any clinically significant medical condition, including cardiovascular abnormalities on ECG or laboratory abnormality that could interfere with the conduct of the study; use of any investigational drug; positive test for drugs or alcohol; or positive test for hepatitis B surface antigen (HBsAg), hepatitis C virus (HCV), or human immunodeficiency virus (HIV). Subjects with RI or ESRD could be excluded for a hemoglobin concentration of <9.0 g/dl. Subjects with RI could be excluded for an acute exacerbation of RI, and subjects with ESRD could be excluded for use of blood purification therapy other than HD.

### Study treatments.

For healthy subjects and those with mild or moderate RI, single 1,000-mg doses of durlobactam and sulbactam were administered via a 3-h i.v. infusion. After assessing safety and PK data for the mild and moderate RI groups, subjects with severe RI received single 500-mg i.v. doses of durlobactam and sulbactam administered via a 3-h i.v. infusion. For subjects with ESRD and HD, 500-mg i.v. doses of durlobactam and sulbactam were administered post-HD and pre-HD via 3-h i.v. infusion with at least a 1-week washout between doses.

### Assessments.

Safety was assessed from reports of adverse events (AEs), vital signs (heart rate, blood pressure, respiratory rate, and oral temperature), physical examination, 12-lead ECG, and clinical laboratory testing (chemistry, hematology, and urinalysis).

Venous blood samples for PK analyses of durlobactam and sulbactam were obtained predose and 0.5, 1, 2, 3, 3.5, 4, 5, 6, 7, 8, 12, 24, 36, and 48 h after the start of infusion for cohorts 1 and 2. An additional 60-h time point was taken for subjects in cohorts 3, 4, and 5 (periods 1 and 2). Urine for PK analyses was collected at the following intervals: predose (a single void within 30 min prior to start of infusion) and 0 to 4, 4 to 8, 8 to 12, and 12 to 24 h after the start of infusion for cohorts 1 and 2. Additional collections at 24 to 36, 36 to 48, and 48 to 60 h were obtained in cohorts 3 and 4. For ESRD subjects, venous samples were collected at 10 and 16 h, in addition to the normal schedule of venous samples for PK analysis. Dialysis was started approximately 1 h after the end of infusion. If the HD session required more than 7 h after the start of infusion, a final sample was collected at the end of the HD session. Dialysate was collected for PK analyses at approximately 4 to 5, 5 to 6, 6 to 7, and from 7 h after the start of infusion until the end of HD.

### Pharmacokinetic analysis.

Pharmacokinetic parameters were calculated using noncompartmental analysis with Phoenix WinNonlin version 6.3 (Certara USA, Inc., Princeton, NJ). Actual sample collection times and infusion durations were used, with no adjustments where infusion was interrupted. ln-transformed dose-normalized durlobactam and sulbactam plasma PK parameters (area under the plasma concentration versus time curve from time 0 to time of last quantifiable concentration after dosing [AUC_0–last_/dose], AUC extrapolated to infinity [AUC_0–inf_/dose], and *C*_max_/dose) from subjects with mild, moderate, and severe RI and those with ESRD on HD were compared to the corresponding parameters in healthy subjects using analysis of variance (SAS version 9.3; SAS Institute, Cary, North Carolina, US), with cohort modeled as a fixed effect. Estimates of differences were back transformed to provide a ratio of the geometric least-squares means (LSM) and the corresponding 90% confidence intervals (CIs). A similar comparison was performed for the ESRD group to compare these same PK parameters by hemodialysis (period 2, dialysis initiated 1 h after the end of infusion) against those with no dialysis (period 1, dose administered after the end of dialysis). The ANOVA included cohort modeled as a fixed effect and subject as a random effect. The relationship between PK parameters and estimated renal function (both eGFR and CL_CR_) was modeled for healthy subjects and subjects with RI by regression to provide estimates of correlation and the slope and corresponding 95% CI to guide dosing recommendations for subjects with RI.

### Statistical analysis.

Continuous data were summarized with descriptive statistics. For continuous PK parameters, geometric mean with the associated 95% CIs and the geometric between-subject coefficient of variation (CV%) were determined. Categorical data were summarized with frequencies and percentages. Discrete time-related PK parameters were summarized using median, minimum, and maximum.

## Supplementary Material

Supplemental file 1
