# Supplementary material for: Pharmacokinetics, Safety, and Tolerability of Intravenous Durlobactam and Sulbactam in Subjects with Renal Impairment and Healthy Matched Control Subjects
Source: Antimicrob Agents Chemother. 2019 Aug 23;63(9):e00794-19. doi: 10.1128/AAC.00794-19 (PMC6709459; doi:10.1128/AAC.00794-19)
Supplement: Supplemental file 1 [file AAC.00794-19-s0001.pdf]

Data Supplement

Pharmacokinetics, Safety, and Tolerability of Intravenous Durlobactam and Sulbactam in Subjects with Renal Impairment and Healthy Matched Control Subjects

Supplemental Table 1. Summary of ANOVA for Durlobactam and Sulbactam PK Parameters Among RI Subjects

Parameter	Cohort	Geometric LSMean	% Ratio of Geometric LSMean (%)	90% Confidence Intervals for Ratio
**Durlobactam**				
C_{max}/Dose (μg/mL/mg)	2 (test)<br>1 (reference)	0.0333<br>0.0270	124	101.7, 150.0
C_{max}/Dose (μg/mL/mg)	3 (test)<br>1 (reference)	0.0387<br>0.0270	144	118.3, 174.4
C_{max}/Dose (μg/mL/mg)	4 (test)<br>1 (reference)	0.0511<br>0.0270	190	157.5, 228.5
C_{max}/Dose (μg/mL/mg)	5 (test)<br>1 (reference)	0.0572<br>0.0270	212	172.9, 260.6
$AUC_{0\text{-}last}$/Dose (h*μg/ml/mg)	2 (test)<br>1 (reference)	0.151<br>0.109	139	108.1, 177.9
$AUC_{0\text{-}last}$/Dose (h*μg/ml/mg)	3 (test)<br>1 (reference)	0.209<br>0.109	192	149.9, 246.7
$AUC_{0\text{-}last}$/Dose (h*μg/ml/mg)	4 (test)<br>1 (reference)	0.404<br>0.109	372	293.2, 472.6
$AUC_{0\text{-}last}$/Dose (h*μg/ml/mg)	5 (test)<br>1 (reference)	0.557<br>0.109	513	394.5, 667.4
$AUC_{0\text{-}inf}$/Dose (h*μg/ml/mg)	2 (test)<br>1 (reference)	0.151<br>0.110	137	105.5, 178.1
$AUC_{0\text{-}inf}$/Dose (h*μg/ml/mg)	3 (test)<br>1 (reference)	0.209<br>0.110	190	146.2, 246.8
$AUC_{0\text{-}inf}$/Dose (h*μg/ml/mg)	4 (test)<br>1 (reference)	0.404<br>0.110	368	286.0, 473.1
$AUC_{0\text{-}inf}$/Dose (h*μg/ml/mg)	5 (test)<br>1 (reference)	0.560<br>0.110	510	386.8, 671.3
**Sulbactam**				
C_{max}/Dose (μg/mL/mg)	2 (test)<br>1 (reference)	0.0221<br>0.0170	130	103.2, 164.1
C_{max}/Dose (μg/mL/mg)	3 (test)<br>1 (reference)	0.0271<br>0.0170	159	126.4, 200.9
C_{max}/Dose (μg/mL/mg)	4 (test)<br>1 (reference)	0.0405<br>0.0170	238	190.5, 296.9

C_{max}/Dose (μg/mL/mg)	5 (test)<br>1 (reference)	0.0473<br>0.0170	278	217.6, 354.9
$AUC_{0\text{-}last}$/Dose (h*μg/ml/mg)	2 (test)<br>1 (reference)	0.0853<br>0.0628	136	95.8, 193.0
$AUC_{0\text{-}last}$/Dose (h*μg/ml/mg)	3 (test)<br>1 (reference)	0.126<br>0.0628	200	141.1, 284.5
$AUC_{0\text{-}last}$/Dose (h*μg/ml/mg)	4 (test)<br>1 (reference)	0.273<br>0.0628	435	310.7, 608.3
$AUC_{0\text{-}last}$/Dose (h*μg/ml/mg)	5 (test)<br>1 (reference)	0.561<br>0.0628	894	617.2, 1293.7
$AUC_{0\text{-}inf}$/Dose (h*μg/ml/mg)	2 (test)<br>1 (reference)	0.0858<br>0.0630	136	95.6, 193.8
$AUC_{0\text{-}inf}$/Dose (h*μg/ml/mg)	3 (test)<br>1 (reference)	0.126<br>0.0630	200	140.5, 284.9
$AUC_{0\text{-}inf}$/Dose (h*μg/ml/mg)	4 (test)<br>1 (reference)	0.273<br>0.0630	433	308.9, 608.2
$AUC_{0\text{-}inf}$/Dose (h*μg/ml/mg)	5 (test)<br>1 (reference)	0.575<br>0.0630	912	628.1, 1324.9

Supplemental Table 2. Summary of ANOVA for Durlobactam and Sulbactam PK Parameters Among ESRD Subjects

Parameter	Cohort	Geometric LSMean	% Ratio of Geometric LSMean (%)	90% Confidence Intervals for Ratio
**Durlobactam**				
C_{max}/Dose (µg/mL/mg)	Pre-HD (test)<br>Post-HD (reference)	0.0421<br>0.0572	73.6	60.8, 89.1
$AUC_{0\text{-}last}$/Dose (h*µg/ml/mg)	Pre-HD (test)<br>Post-HD (reference)	0.238<br>0.557	42.8	34.1, 53.7
$AUC_{0\text{-}inf}$/Dose (h*µg/ml/mg)	Pre-HD (test)<br>Post-HD (reference)	0.235<br>0.522	45.1	34.1, 59.6
**Sulbactam**				
C_{max}/Dose (µg/mL/mg)	Pre-HD (test)<br>Post-HD (reference)	0.0385<br>0.0473	81.3	65.2, 101.4
$AUC_{0\text{-}last}$/Dose (h*µg/ml/mg)	Pre-HD (test)<br>Post-HD (reference)	0.244<br>0.561	43.6	31.3, 60.7
$AUC_{0\text{-}inf}$/Dose (h*µg/ml/mg)	Pre-HD (test)<br>Post-HD (reference)	0.247<br>0.536	46.1	29.8, 71.5
